# Time-Resolved Analysis of Candidate Gene Expression and Ambient Temperature During Bud Dormancy in Apple

**DOI:** 10.3389/fpls.2021.803341

**Published:** 2022-01-17

**Authors:** Janne Lempe, Andreas Peil, Henryk Flachowsky

**Affiliations:** Julius Kühn Institute (JKI) – Federal Research Centre for Cultivated Plants, Institute for Breeding Research on Fruit Crops, Dresden, Germany

**Keywords:** apple, dormancy, temperature, *DAM* genes, endodormancy, ecodormancy

## Abstract

Winter dormancy – a period of low metabolic activity and no visible growth – appears as an adaptation to harsh winter conditions and can be divided into different phases. It is tightly controlled by environmental cues, with ambient temperature playing a major role. During endodormancy, a cultivar-specific amount of cold needs to be perceived, and during ecodormancy, heat hours accumulate before bud burst and anthesis in spring. Expression analysis, performed in several key fruit tree species, proved to be very useful in elucidating the molecular control of onset and release of dormancy. However, the time resolution of these experiments has been limited. Therefore, in this study, dense time-series expression analysis was conducted for 40 candidate genes involved in dormancy control, under the cool-temperate climate conditions in Dresden. Samples were taken from the cultivars ‘Pinova’ and ‘Gala,’ which differ in flowering time. The set of candidate genes included well-established dormancy genes such as *DAM* genes, *MdFLC-like, MdICE1, MdPRE 1*, and *MdPIF4.* Furthermore, we tested genes from dormancy-associated pathways including the brassinosteroid, gibberellic acid, abscisic acid (ABA), cytokinin response, and respiratory stress pathways. The expression patterns of well-established dormancy genes were confirmed and could be associated with specific dormancy phases. In addition, less well-known transcription factors and genes of the ABA signaling pathway showed associations with dormancy progression. The three ABA signaling genes *HAB1*_*chr15, HAI3*, and *ABF2* showed a local minimum of gene expression in proximity of the endodormancy to ecodormancy transition. The number of sampling points allowed us to correlate expression values with temperature data, which revealed significant correlations of ambient temperature with the expression of the *Malus domestica* genes *MdICE1, MdPIF4*, *MdFLC-like*, *HAB1chr15*, and the *type-B cytokinin response regulator BRR9*. Interestingly, the slope of the linear correlation of temperature with the expression of *MdPIF4* differed between cultivars. Whether the strength of inducibility of *MdPIF4* expression by low temperature differs between the ‘Pinova’ and ‘Gala’ alleles needs to be tested further.

## Introduction

In deciduous fruit trees such as apple (*Malus* × *domestica* Borkh.), phases of high metabolic activity, growth, and reproduction alternate with phases of no growth and low levels of activity, called dormancy. During dormancy, all vulnerable tissues are either removed or protected by very robust bud scales, which allows surviving harsh winter conditions. The cycles of activity and dormancy need to be synchronized well with environmental conditions in order to maximize survival during winter, optimize the timing of reproduction and yield, and maximize the length of growing seasons (Cooke et al., 2012). Which environmental cues regulate onset, progression, and release of dormancy in deciduous fruit trees is not entirely resolved, but ambient temperature and photoperiod appear to play major roles (Heide, 2008; Cooke et al., 2012; Maurya and Bhalerao, 2017). In contrast to most fruit tree species, *M.* × *domestica* is not sensitive to photoperiod and thus uses ambient temperature as a major environmental cue to control the timing of dormancy (Heide and Prestrud, 2005; Tanino et al., 2010; Cooke et al., 2012; Singh et al., 2017). Whether light quality serves as an additional cue needs to be determined.

Winter dormancy consists of three distinct phases, namely, paradormancy, endodormancy, and ecodormancy (Lang, 1987). During paradormancy, the termination of shoot growth is induced by other parts of the growing plant (Singh et al., 2017). However, dormant buds are still competent to leaf out, when disconnected from internal inhibitory signals from the tree and when grown under suitable conditions (Saure, 1985). During endodormancy, internal conditions prevent buds from leafing out, even if disconnected from the tree and placed under suitable growth conditions. In order to transit from endodormancy to ecodormancy, a genotype-specific amount of cold needs to be perceived, which is called chilling requirement (CR) (Coville, 1920). Freezing tolerance is highest during endodormancy, where water is bound and unfreeze (Erez et al., 1990; Faust et al., 1991). After the transition to ecodormancy, bud behavior is controlled predominantly by external conditions. If temperature accumulation is sufficiently high, buds will leaf out. For the phase transition from ecodormancy to bud break, it is heated, which needs to accumulate (heat requirement, HR). Several temperature-based models have been developed to measure the amounts of cold and heat that are required to progress through dormancy and allow to forecast the timing of bud break (Fadon et al., 2020). The most commonly used models are the chilling hours/Weinberger-Eggert model (Weinberger, 1950), the Utah model (Richardson et al., 1974), and the dynamic model (Fishman et al., 1987a,b; Erez et al., 1990). Model comparisons suggest that the goodness of performance of each model depends on the climatic region and the plant species it is applied to Luedeling et al. (2009); Chmielewski et al. (2012). The most commonly used model for heat quantification is the growing degree hour (GDH) model by Anderson et al. (1986). How chill and heat units are perceived and how their accumulation is resolved molecularly are still open questions.

In regions of the mild climate, chilling accumulates very slowly, leading to a prolonged phase of endodormancy, which may overlap with ecodormancy (Saure, 1985; Malagi et al., 2015). In temperate climate zones, there is sufficient chilling, and the phase transition to ecodormancy occurs at winter times when low temperature alone is sufficient to inhibit further growth. Global warming poses significant challenges to the progression of dormancy that are not universal but rather specific to the perceived environmental conditions. In mild climates, not enough chill units accumulate, which leads to irregular budburst and to lower crop yield (Erez, 2000). In the temperate zone, warm springs cause a shift to earlier bud break, with the consequence of the high risk of flower damage by early spring frost (Chmielewski et al., 2004; Estrella et al., 2007). Therefore, it is of high interest to unravel the molecular mechanisms of winter dormancy, which will allow developing tools for breeding apple cultivars, which are resilient to climate change.

In recent years, progress has been made on resolving the molecular mechanisms of dormancy control (Falavigna et al., 2019). *Dormancy-Associated MADS-Box* (*DAM*) genes were the first genes identified from the *evergrowing* mutant in peach (Bielenberg et al., 2008), and later also in other fruit-bearing species of the *Rosaceae*, such as plum, cherry, Japanese apricot, apple, and pear (Mimida et al., 2015; Rothkegel et al., 2017; Zhao et al., 2018; Vimont et al., 2019; Quesada-Traver et al., 2020).

*Short Vegetative Phase* (*SVP*) genes are structurally similar to *DAM* genes and are also important for dormancy control. Lines with transgenically downregulated levels of *MdDAM* and *MdSVP* genes are not able to get into the dormant state (Wu et al., 2017, 2021; Moser et al., 2020). Interestingly, *MdDAM*, *MdSVP*, and *Md Flowering Locus C-like* (*MdFLC*-like) genes form multimeric complexes that bind to specific DNA regions (Falavigna et al., 2021).

Since functional verification by transgenic lines is difficult, gene expression analysis has been very important to get insight into genes and pathways involved in dormancy control. Several RNAseq studies have been conducted in apple, peach, cherry, and apricot (Falavigna et al., 2014; Takeuchi et al., 2018; Artlip et al., 2019; Vimont et al., 2019; Moser et al., 2020; Yu et al., 2020; Zhu et al., 2020). The strict filtering strategy of Takeuchi et al. (2018) revealed a small set of genes being involved in endodormancy phase transition and showed expression correlation with ambient temperature under laboratory conditions. Among them were *MdFLC-like* and *PHYTOCHROME-INTERACTING FACTOR 4* (*MdPIF4*), both have been associated with a temperature-sensitive expression before. In *Arabidopsis thaliana*, PIFs interact with light receptors and mediate growth responses by modifying the chromatin organization (Kumar et al., 2012; Willige et al., 2021). *MdFLC-like* in apple has been shown to be upregulated on cold treatment and may confer a growth-inhibiting effect (Porto et al., 2015; Nishiyama et al., 2021).

Although whole-genome expression analysis has revealed important insight, the time resolution of sampling dates is very limited due to the long time span that needs to be covered. A common strategy has been to sample once a month or to sample after plants have perceived a defined amount of chill hours (CH), which results in a total of four to eight sampling dates across winter (Moser et al., 2020; Yu et al., 2020; Falavigna et al., 2021). Vimont et al. (2019) also sampled every month; however, they improved the study considerably by selectively increasing sampling density in the weeks before phase transition (Vimont et al., 2019). By doing so, they increased the sampling density to biweekly for a few weeks. Yet, higher sampling densities are required to adequately capture the dynamic process of dormancy and the transitions between phases. Therefore, to unravel the molecular mechanisms of winter dormancy, a gene expression analysis of selected dormancy candidate genes was carried out. In this study, the high sampling density allowed capturing the dynamic process of dormancy and the transition between its individual phases. Buds of the two apple cultivars ‘Gala’ and ‘Pinova’ were collected from a commercial orchard in the Southeast of Germany, under the cool-temperate climate conditions in Dresden (Germany), which is located in the transition zone to the continental climate. The expression patterns of these genes were determined in densely spaced samples, which were taken weekly, covering the complete time span of winter dormancy. The dense sampling allowed us to identify specific expression patterns that were associated either with endodormancy or ecodormancy or patterns that reached across both phases. Correlations of gene expression with temperature under natural conditions provided further evidence for temperature-dependent expression.

## Materials and Methods

### Plant Material

Spur buds of the two *M.*×*domestica* Borkh. cultivars ‘Pinova’ and ‘Gala’ grafted on M9 rootstocks were collected at the Orchard Görnitz in Neusörnewitz, located near Dresden, Germany (latitude 51.14908600295051, longitude 13.54123977860281). Sampling took place every week from September 20, 2017 to April 18, 2018. Per time point and cultivar, four biological replicates were collected with five spur buds for each replicate.

### Timing of Dormancy Phase Transitions

The timing of the phase transition from endodormancy to ecodormancy was determined by scion cuttings. Therefore, extension shoots with a swollen terminal bud were cut every week and placed in a growth chamber at 20°C long days (16 h light) and relative humidity of 60%. Scions were placed in jars containing tap water, which was renewed every week. The developmental stages of generative terminal buds were determined for three replicates. Scions were cut every week from December 12, 2017 to February 14, 2018. The date of transition was defined as the cutting date of scions that under bud break forcing conditions reached the developmental stage BBCH 59 (ballon) after 5 weeks. Bud break and flowering in the field were scored every week during spring 2018 on 10 trees per cultivar.

### Determination of Chilling and Heat Requirements

Hourly temperature data were obtained from the meteorological station located in Coswig at a distance of 3.38 km from the orchard. The accumulation of chilling hours was calculated by counting any hour as one unit if its temperature lies between 0 and 7.2°C (Weinberger, 1950). Chill portions (CP) were calculated by the dynamic model by Fishman et al. (1987a,b); Erez et al. (1990). Heat accumulation was calculated using the ASYMCUR GDH model by Anderson with the base, optimal, and critical temperatures of 4°C, 25°C, and 36°C, respectively (Anderson et al., 1986; Milyaev et al., 2021).

### Sample Preparation, RNA Extraction, and cDNA Synthesis

Immediately after harvesting, bud scales were removed, and samples were frozen on dry ice. Frozen buds were homogenized with the Retsch Mixer Mill MM400 (Retsch, Germany), total RNA was extracted using the InviTrap Spin Plant RNA Mini Kit (Stratec Molecular GmbH, Berlin, Germany), and DNA was removed with the DNA-Free Kit (Life Technologies GmbH, Darmstadt, Germany). cDNA was synthesized with oligo_dT_ primer and 1 μg RNA as template and diluted 1:20 for gene expression analysis.

### Primer Design

Primers were designed with the NCBI Tool Primer-BLAST, combining Primer3 and BLAST with the standard settings and *M. domestica* taxid 3750 as reference organism. To assure specificity to the gene of interest, suggested primer sequences were only considered if they did not target any other sequence with zero to two mismatches. All mRNA target sequences were extracted from the GDDH1.3 Genome (Daccord et al., 2017). To identify *M. domestica* genes that are homologous to the selected *Prunus persica* genes of interest, nucleotide BLAST against the *M. domestica* GDDH1.3 genome was used. Exceptions were sequences for *Md Flowering locus T 2 (MdFT2)*. The sequence of *MdFT2* (HQ424012.1 ‘Pinova’ and HF31588-RA ‘Hanfu line’) was not found in the GDDH1.3 genome by BLAST, and only the sequence of *MdFT1* (HQ424013.1 ‘Pinova,’ *MD12G1262000* ‘Golden Delicious’, HF38537-RA ‘Hanfu line’) was found in the GDDH1.3 genome. Therefore, the primers *MdFT2*-qRT-PCR of Kotoda et al. (2010) were used for PCR. All primer pairs were tested on ‘Pinova’ and ‘Gala’ cDNA by qRT-PCR (95°C for 15 s, 60°C for 30 s, 72°C for 30 s, for 40 cycles), and specificity was confirmed by melting curve analysis. For *PIF4*, primer efficiency was determined for each cultivar *via* concentration series and was very similar for both cultivars (Supplementary Figure 1). A full list of primers is given in Supplementary Table 1. The presence of *MdDAM1* splice variants was tested by PCR on cDNA of four replicates of ‘Pinova’ samples collected between November 1, 2017 and December 13, 2017 at an annealing temperature of 57°C, an extension time of 1 min, and with 40 PCR cycles.

### Gene Expression Analysis With the BioMark HD System

Expression analysis was performed using the BioMark HD high-throughput system with the 96.96 Dynamic Array (Fluidigm, South San Francisco, California, United States). For the winter period of 2017/2018, three biological and two technical replicates of two cultivars at 28 were analyzed with 44 primer pairs (Supplementary Table 1). The 96.96 Dynamic Array Integrated Fluidic Chip assay was performed according to the instructions of the manufacturer and as described by Reim et al. (2020). Expression data were analyzed with the Fluidigm Real-Time polymerase chain reaction (PCR) Analysis 3.1.3 software (Fluidigm, South San Francisco, California, United States). The amplification curve quality check was performed, and three primer pairs and three samples (i.e., 114_rep1_Pinova, 120_rep3_Pinova, and 123_rep1_Gala) were removed from further analysis as their quality score was below the set threshold of 0.65. The linear baseline correction and auto cycle threshold (Ct) determination were used for retrieving CT values. The expression stability of reference genes was validated using RefFinder (Xie et al., 2012). Relative expression values were calculated using the 2^–ΔΔCT^ method, using sample 101 as the calibrator (Pfaffl, 2001). Graphical representation of expression data was performed with ggplot2 using R (Wickham, 2016, R foundation, Vienna, Austria).

### Correlation of Temperature Data With Gene Expression Levels

Mean expression values per time point and cultivar were correlated with temperature data. Therefore, the following temperature values were used: mean daily temperature at the sampling date, and the sum of CH or CP of all days within 2 weeks before sampling was calculated. The correlation matrix was calculated and plotted using the Pearson correlation method with the R package corrplot (Wei and Simko, 2021). Scatter plots and linear regressions were performed using R and ggplot2 (Wickham, 2016).

### Cluster Analysis

*k*-means clustering was performed using the R package “cluster,” and the R package “factoextra” was used to visualize the clusters (Kassambara, 2017; Maechler et al., 2021). The optimal cluster number was determined by considering the within the sum of squares per number of clusters. For iterations, 25 random starting assignments were used, which revealed 25 different clustering results. Of these results, the best one with the smallest sum of squares was chosen.

## Results and Discussion

### Selection of Candidate Genes

A set of 40 candidate genes was selected to determine specific expression patterns during winter dormancy in buds of the two apple cultivars ‘Gala’ and ‘Pinova’ (Supplementary Table 2). These include transcription factors and metabolic enzymes that are clearly or less explicitly associated with dormancy.

The MADS-box transcription factor genes *MdDAM1, MdDAM2, MdDAM4, MdSVPa*, and *MdSVPb* are well known to control the progression of dormancy in fruit trees of the *Rosacea* (Falavigna et al., 2019). The gene *MdFLC-like* also belongs to MADS-box transcription factors and is the closest paralog of the *A. thaliana* flowering time gene *Flowering Locus C (FLC)* (Kumar et al., 2016). It was chosen because *MdFLC-like* was upregulated after cold exposure, and evidence exists for a growth-inhibiting effect, which suggests that *MdFLC-like* is a good candidate for playing a role in dormancy (Porto et al., 2015; Takeuchi et al., 2018; Nishiyama et al., 2021). Additionally, it was located on top of chromosome 9, where quantitative trait loci (QTL) - and genome-wide association studies identified loci controlling bud break in apple (Vimont et al., 2019; Celton et al., 2011; Allard et al., 2016; Urrestarazu et al., 2017). Additionally, the four AP2/ethylene-responsive transcription factor genes *Md Early Bud Break1* (*MdEBB1), APETALA 2 on chromosome 7(AP2chr7), APETALA 2/Ethylene-Responsive Factor 113 (AP2_ERF113)*, and *Ethylene-Responsive Factor on chromosome 4 (ERFchr4)* described below were also included. *EBB1* has been identified in poplar to play a role in dormancy release (Yordanov et al., 2014) and was associated with bud break in apples (Wisniewski et al., 2015). In pear, correlations of *PpEBB1* with *cyclin D3* were observed, and the hypothesis was put forward that *EBB1* could regulate bud break by activating cell cycle regulator genes to reinitiate cell division (Tuan et al., 2016). *AP2chr7* and *ERFchr4* were differentially expressed in *MdDAM1*-silenced transgenic apple trees and changed expression during the course of dormancy (Moser et al., 2020). *AP2_ERF113* showed temperature-sensitive expression variation during dormancy, similar to the gene *PIF4*, the *MYC2-like* transcription factor *MD14G1126900 (MYC2-like)*, *F-box* transcription factor *MD11G1009500 (MdFbox), Peroxidase 42-like MD10G1321200 (Md_peroxidase)*, and *4-Coumarate coenzyme A ligase 5 MD13G1257800 (CoA_ligase)* (Takeuchi et al., 2018). *MdbZIP* and the NAC-like transcription factor NAM were included because both are differentially expressed in *MdDAM1-*silenced trees and varied expression during dormancy progression (Moser et al., 2020). The transcription factor gene *Inducer of CBF Expression1 (ICE1)* was found in a pear to be associated with endodormancy (Takemura et al., 2015) and was identified as the candidate gene in previous QTL studies investigating the timing of bud break, similar to *PACLOBUTRAZOL RESISTANCE 1 (MdPRE1*) (Miotto et al., 2019). Additional to *MdPRE1*, two more members of the Brassinosteroid pathway were selected, namely, *Brassinosteroid-6-Oxidase 2 (BR6OX)* and the receptor Kinase inhibitor *BKI1*. *BR6Ox* and *BKI1* were chosen because both showed differential expression in dormancy-related RNA-seq experiments in cherry, peach, and apricot (Vimont et al., 2019; Yu et al., 2020). Of the gibberellic acid pathway, two *gibberellin 3-oxidases 1* (*GA3Ox1, MD08G1138400, MD15G1116300*) and one *gibberellin 2-oxidase 8* (*GA2Ox8, MD11G1225400*) were included because close homologs were differentially expressed in cherry, peach, and apricot during dormancy progression (Vimont et al., 2019; Yu et al., 2020). From the abscisic acid (ABA) pathway, the following four transcription factor genes were included: *ABSCISIC ACID RESPONSIVE ELEMENTS-BINDING FACTOR 2* (*ABF2, MD15G1081800), ABF2* on chromosome 8 *(ABFchr8), (MD08G1099600), ABA-Insensitive 5 (ABI5) on chromosome 12,(ABI5chr12, MD12G1024300), and ABI5* on chromosome 14 (*ABI5chr14, MD14G1021600*). These were included because they were found to control a cluster of differentially expressed genes during dormancy in cherry (Vimont et al., 2019). Further members of the ABA pathway that were included in this study were the genes *Group A protein type 2C phosphatases HAB1chr15 (MD15G1212000), HAB1chr2 (MD02G1084600)*, and the *HIGHLY ABA-INDUCED PP2C Gene 3* (*HAI3, MD06G1106000)* and the epoxidase *ABA1*, which catalyzes the first step of ABA biosynthesis (*MD02G1318300, MD07G1003000*). This group of genes was differentially expressed during dormancy progression in peach and apricot (Yu et al., 2020). From the cytokinin signaling pathway, the type-B cytokinin response regulators *BRR1, 7, 8, 9*, and *10* were included in this study, as they were found to play a role in the endodormancy to ecodormancy transition in apple previously (Cattani et al., 2020). Not only hormones but also respiratory stress has been proposed to play a role in dormancy release (Vimont et al., 2019); therefore, the gene *Respiratory burst oxidase protein F (RBOH, MD14G1211700)* was included, which is differentially expressed in cherry, peach, and apricot, as well as *Md_peroxidase*, mentioned above).

### Timing of the Endodormancy to Ecodormancy Transition With Relation to Cold and Heat Requirements

To be able to associate expression patterns with the timing of dormancy, the transition of endodormancy to ecodormancy was phenologically determined by scion cuttings and the end of dormancy by scoring bud break in the field (Lang, 1987). During winter 2017/2018, the transition of endodormancy to ecodormancy occurred on January 17 for ‘Pinova’ and on January 31 for ‘Gala’ (Supplementary Figure 2). Until January 17, 2018, trees had received 72.9 CP or 1,344 CH (Supplementary Figure 3). Therefore, the CR for ‘Pinova’ 2018 was 72.9 CP or 1,344 CH. Slightly higher was the CR for ‘Gala,’ 83.9 CP or 1,552 CH, which was reached on January 31. Bud break occurred between April 4 and April 11, 2018 in both cultivars, after an accumulation of 2,619–2,915 GDH.

### Gene Expression Patterns

**FIGURE 1:**
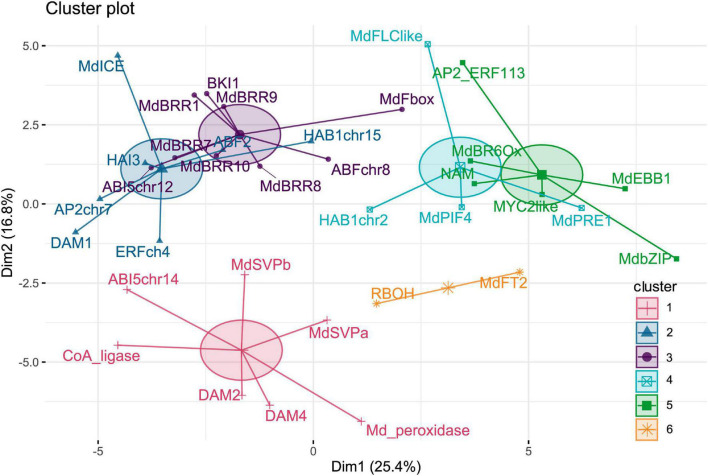
Clusters of genes determined by *k*-means clustering of gene expression patterns. A total of 35 genes were clustered into six clusters according to their gene expression patterns across all samples.

The time points of sample collections were chosen to cover all dormancy phases from the end of summer (September 20) to spring (April 18). Dense sampling allows monitoring expression changes that occur during short time spans. The expression pattern of 40 selected genes was surveyed, and for 35 genes, distinct patterns of expression were determined, and five genes did show no expression. To group genes by overall expression pattern across all time points, *k*-means clustering analyses were performed. Expression patterns were clustered into six different clusters (Figure 1). Cluster 1 and cluster 6 are distinct with good separation, whereas clusters 2 and 3, as well as clusters 4 and 5, are less distant from each other.

**FIGURE 2:**
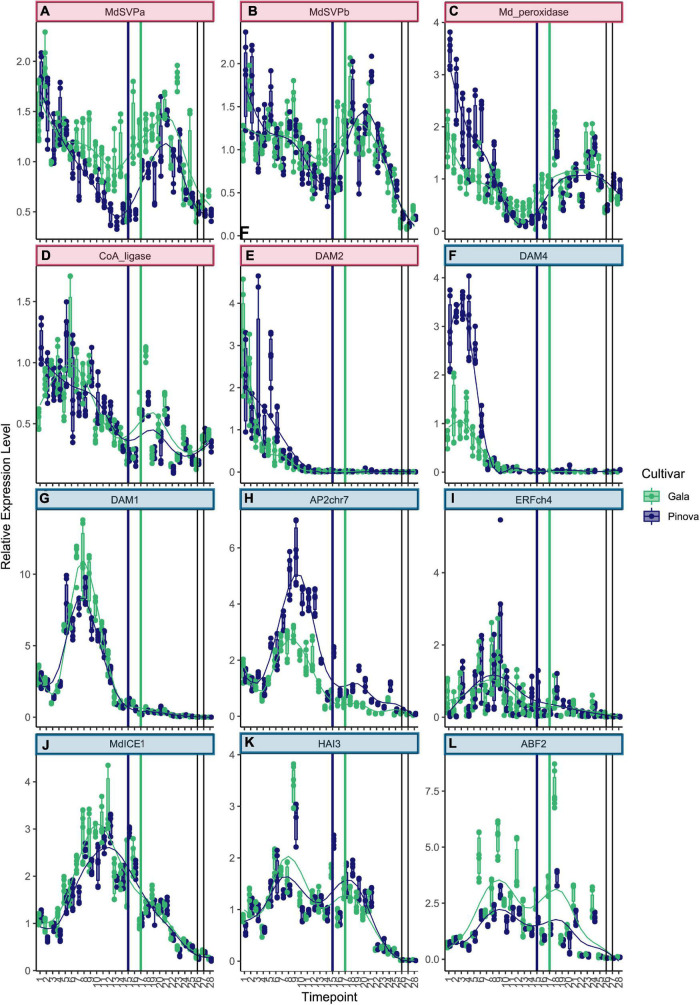
Expression patterns of genes belonging to clusters 1 and 2. **(A)** *MdSVPa, MdSVPb* **(B)**, and *Md_peroxidase* **(C)** were highly expressed at the beginning of dormancy, expression levels dropped to a local minimum shortly before the endodormancy to ecodormancy phase transition, and raised again to peak during ecodormancy. **(D)** *CoA-ligase* showed a similar pattern. **(E)** *MdDAM2* expression levels were highest at the first three sampling dates during early fall. **(F)** Expression of *MdDAM4* peaked on October 18 in both cultivars, levels were higher in ‘Pinova’ (blue) compared with ‘Gala’ (green). **(G)** *MdDAM1* peaked on November 23, similar to *AP2chr7* **(H)** and *ERFchr4* **(I)**. **(J)** The expression curve of *MdICE* was broader. **(K)** The expression pattern of *HAI3* and *ABF2* **(L)** showed two peaks. Cluster number is indicated by colored box harboring the gene name. The colors of the boxes match the cluster colors in Figure 1. Blue and green dots represent ‘Pinova’ and ‘Gala’ samples, respectively. Vertical lines mark the transition from ecodormancy to endodormancy of ‘Pinova’ (blue, January 17) and ‘Gala’ (green, January 31, 2018). Gray vertical lines indicate the time of bud break. Numbers on the *x*-axis indicate sample collection dates specified in Supplementary Table 3.

**FIGURE 3:**
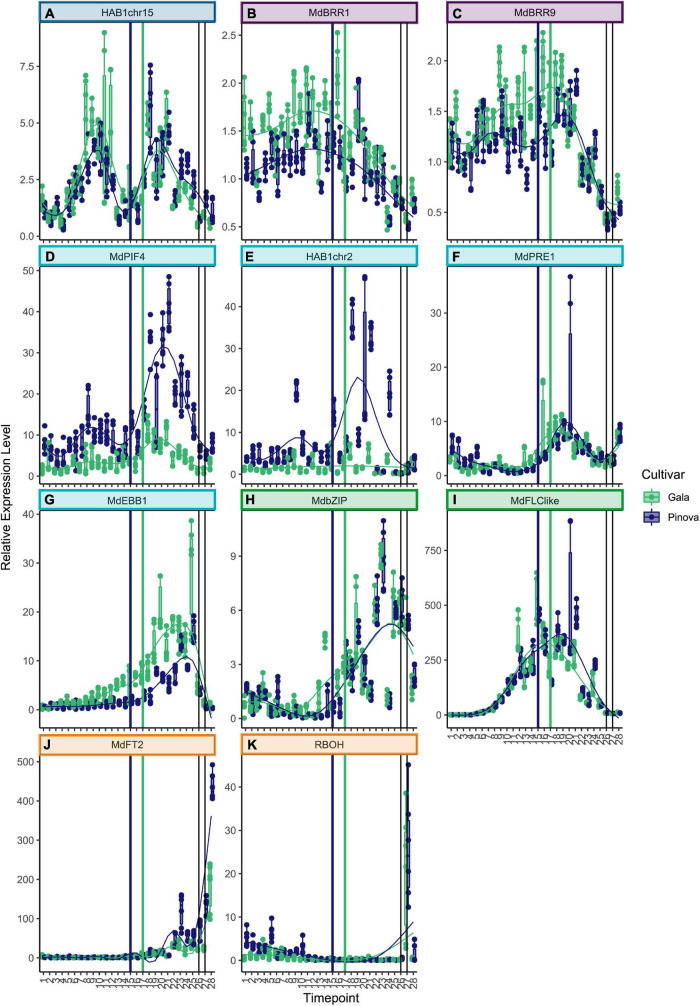
Expression patterns of genes belonging to clusters 2–6. **(A)** *HAB1chr15* showed two similarly strong peaks of expression, on December 6 and February 14, and the local minimum was on January 10, 1 week before the ecodormancy to endodormancy transition. **(B)** *MdBRR1* and *MdBRR9* **(C)** were expressed broadly across dormancy and declined before bud break. **(D)** Expression of *MdPIF4* as well as for *HAB1chr2* **(E)** showed two peaks of which the first peak was lower than at the second peak, and ‘Pinova’ expression levels were higher compared to ‘Gala.’ **(F)** The expression of *MdPRE1* was low during endodormancy and showed a maximum of expression on February 7 and 14. **(G)** The expression levels of *MdEBB1* and *MdbZIP* peaked slightly later, on March 7 and March 14 **(H)**. **(I)** *MdFLC-like* was expressed broadly and it peaked later in ‘Pinova’ compared with ‘Gala.’ **(J)** *RBOH* and *MdFT2* **(K)** were not expressed during dormancy; however, they were strongly induced on bud break. The color scheme is identical to the one in Figure 2.

Genes that belong to cluster 1 were highly expressed at the very beginning of dormancy (Figures 2A–E. See Supplementary Table 3 for collection dates). The two known dormancy-related *SVP* genes *MdSVPa* and *MdSVPb* belong to this first cluster (Figures 2A,B). Their expression levels were high at the beginning of dormancy, dropped to a local minimum during the ecodormancy to endodormancy transition, and increased again to reach a second maximum of expression during mid of ecodormancy. The genes *CoA_ligase* and *Md_peroxidase* showed a pattern of expression that is very similar to *MdSVPa* and *MdSVPb* (Figures 2C,D). Difference in expression between the two cultivars ‘Gala’ and ‘Pinova’ was observed for *MdSVPa*, which showed stronger expression in ‘Gala’ compared with ‘Pinova’, especially during the phase transition and during ecodormancy. The known dormancy-associated MADS-box gene *MdDAM2* also belongs to cluster 1, with high expression levels at the beginning of dormancy (Figure 2E). Less specific was the expression pattern of *ABI5chr14*, which also belongs to cluster 1 (Supplementary Figure 4). Genes of cluster 2 increased expression levels at first and showed a local maximum of expression during endodormancy (Figures 2F–L, 3A). *MdDAM4* and *MdDAM1* belong to cluster 2 and both show a single peak of expression during endodormancy. The peaks of expression occurred progressively, earlier in *MdDAM4* than in *MdDAM1*. A similar pattern was observed for *AP2chr7* and *ERFchr4*. Both genes are members of the ERF/AP2 transcription factor family that are involved in transcriptional control of stress responses and are less well known for playing a role in apple dormancy. ‘Pinova’ expression levels were higher in *MdDAM4* and *AP2chr7* and lower in *MdDAM1* compared with ‘Gala’; however, no shift in the timing of expression was observed. The MADS-box transcription factors *MdSVPa, MdSVPb, MdDAM1, MdDAM4*, and *MdFLC-like* (Figure 3I) have been shown to form multimeric complexes (Falavigna et al., 2021). In this study, we observed overlapping expression patterns of these genes, which may indicate that multimeric complexes including *MdSVPa/b-MdDAM1* or *MdDAM4*, and complexes including *MdSVPa/b* and *MdFLC-like* could potentially be built during time spans of overlapping expression. For *DAM1* in pear, it was shown that alternative splicing variants exist (Li et al., 2021). Since alternative splicing is another mechanism that occurs in relation to temperature-dependent regulation, we also assessed whether splice variants of *MdDAM1* were present in our samples between November 1 and December 22, 2017; however, we did not find evidence for any splice variant of *MdDAM1* in our samples (Supplementary Figure 5). The expression pattern of *MdICE1* (Figure 2J) showed a broader single peak of expression. Expression levels started to rise during endodormancy, were highly expressed during phase transition, declined continuously during ecodormancy, and reached low levels before bud break. The expression peaks occurred approximately 1 week later in ‘Pinova’ compared with ‘Gala.’ In *A. thaliana, ICE1* is an inducer of the cold acclimation pathway that leads to freezing tolerance. Although freezing tolerance overlaps with endodormancy, it appears to be independent of endodormancy (Bilavcik et al., 2015). Furthermore, evidence exists that *ICE1* can activate *FLC* expression (Lee et al., 2015). Since the expression curve of *MdICE1* peaked earlier than that of *MdFLC-like, MdICE1* may also induce expression of *MdFLC-like.* The genes *HAI3*, *ABF2*, and *HAB1chr15* also belong to cluster 2 (Figures 2K,L, 3A). These three genes showed an expression peak during endodormancy, reduced expression, and a local minimum of expression 2–3 weeks before the phase transition to ecodormancy. A second local maximum of expression occurred during ecodormancy. The expression of *ABF2* showed high variation between sampling weeks and was strongly expressed in ‘Gala’ compared with ‘Pinova’ (Figure 2K). Since *HAB1chr15*, *HAI3*, and *ABF2* are all parts of the ABA signaling pathway, these results clearly suggest involvement of ABA signaling during endodormancy and ecodormancy and during phase transition, when expression levels were low. Less distinct were the expression patterns of genes belonging to cluster 3 (Figures 3B,C and Supplementary Figures 4B–H). *MdBRR1* and *MdBRR9* show a broad expression curve across dormancy. ‘Gala’ samples showed higher expression compared with ‘Pinova’ in both genes. Less specific expression patterns, however, certainly expressed during dormancy and with reduced levels before bud break was observed for the genes *ABFchr8, MdFbox, BKI1, MdBRR7, MdBRR8, MdBRR10*, and *ABI5chr12* (Supplementary Figures 4B–H). Genes that belong to cluster 4 show predominant expression during ecodormancy. The genes *MdPIF4*, *HAB1chr2, MdPRE1*, and *MdEBB1* belong to this cluster (Figures 3D–G). *MdPIF4* and *HAB1chr2* showed a small peak of expression during endodormancy and a larger expression peak during ecodormancy. For both genes, expression levels were higher in ‘Pinova’ compared with ‘Gala’. *MdPRE1* and *MdEBB1* had a single peak in their expression pattern during ecodormancy. Cluster 4 comprises expression patterns with a single broader peak that is not lowered during phase transition. *MdbZIP* and *MdFLC-like* belong to this cluster (Figures 3H,I), as well as the following genes with less clear expression patterns: *AP2_ERF113, NAM, MYC2like*, and *MdBR6Ox* (Supplementary Figures 4I,L). The expression levels of *NAM* were much higher in ‘Gala’ compared with ‘Pinova’ throughout dormancy progression. Cluster 6 is a distinct cluster and comprises the two genes *MdFT2* and *RBOH* (Figures 3J,K). Both genes were expressed after dormancy and were associated with bud break.

No expression could be detected for the three genes involved in GA metabolism, two *GA3 oxidases*, one *GA2 oxidase*, and for the two copies of the ABA biosynthesis genes *ABA1* (*MD02G1318300, MD07G1003000*).

These results allow us to speculate on the expression differences between ‘Gala’ and ‘Pinova’ that could potentially cause the difference in dormancy phase transition between these cultivars. Prominent differences between cultivars can be found in the amplitude of expression levels rather than in a time shift. The observed expression pattern also indicates a reduction of expression during endodormancy to ecodormancy transition in several genes. Whether the incomplete reduction of *MdSVPa* expression in Gala during phase transition has an effect on the timing of phase transition could be tested in further experiments.

### Correlation of Gene Expression With Ambient Temperature

Dormancy progression highly depends on ambient temperatures – on chill unit accumulation during endodormancy and on the accumulation of heat units during ecodormancy. To gain insight into the mechanism that allows a temperature-dependent progression of the dormancy cycle, it was tested whether mean expression values of any gene in our field-collected samples correlate with temperature values (Supplementary Figure 6). Strong correlations with environmental temperature indicate the influence of temperature on the control of gene expression.

**FIGURE 4:**
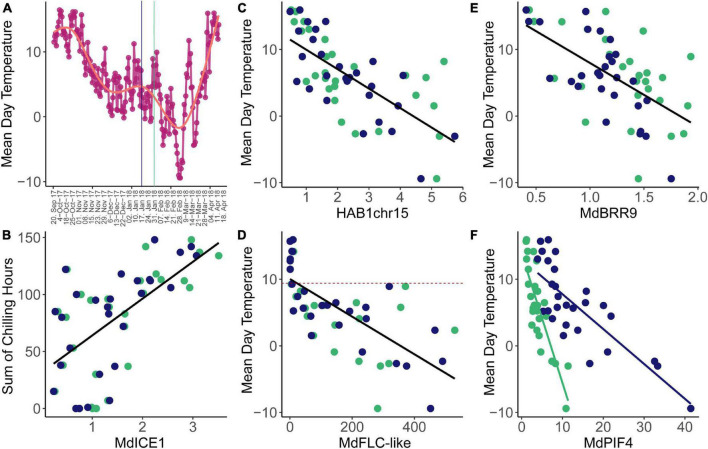
Correlations observed between expression levels and temperature traits. **(A)** Mean daily temperature varied from the first to the last time point of sampling. Vertical lines indicate the timing of the transition from endodormancy to ecodormancy as in Figures 1–3. **(B)** *MdICE1* mean expression levels per time point correlated significantly with the amount of CH that accumulated during the 2 weeks before sampling. **(C)** *HAB1chr16* mean expression levels were highly correlated with mean daily temperature, similar to *MdFLC-like* **(D)** and *MdBRR9* **(E)**. The dashed lines indicate the mean daily temperature of 9.2°C **(D)**. **(F)** The slopes of the linear correlation differ between the two genotypes in *MdPIF4* differed. Blue and green dots represent ‘Pinova’ and ‘Gala’ samples, respectively.

When using accumulated temperature data – accumulated GDH, accumulated CH, or CP – as an environmental variable, correlation analysis did not reveal any meaningful correlations. In order to be able to still use chilling as an environmental variable, we reduced the interval of temperature accumulation to shorter intervals. The most meaningful observed correlations were correlations with the sum of chill units that accumulated 2 weeks before sampling, as well as with mean daily temperature at sampling date (Figure 4A). Using mean daily temperature at the date of sampling for correlation analysis will likely not capture processes that rely on longer time spans. In this study, we expected to identify genes that are able to react to temperature change rather promptly. We imagined dormancy control to occur at multiple time scales and also that the successive progression of several short-term temperature-sensitive events could potentially underlie the observed long-term dormancy progression. Expression of *MdICE1* was positively correlated with CH and showed the highest correlation coefficient of *R* = 0.617 (*p* < 0.05) (Figure 4B). This finding is in line with the knowledge of *ICE1* (*A. thaliana)* being an upstream transcription factor that targets cold-responsive genes conferring freezing tolerance (Gilmour et al., 1998). The highest correlation of expression with mean daily temperature showed *HAB1chr15*, followed by *MdFLC-like, MdBRR9*, and *MdPIF4.* All four genes are negatively correlated with higher expression at lower temperatures (Figures 4C–F). The expression pattern of *HAB1chr15* showed two peaks, which interestingly coincided very well with the lowest mean daily temperatures, resulting in the highest correlation coefficient with a mean daily temperature of all investigated genes (*R* = −0.754, *p* < 0.05). The expression of *MdFLC-like* was also highly correlated with mean daily temperature (*R* = −0.680, *p* < 0.05). Interestingly, *MdFLC-like* was only expressed when the mean daily temperature was below 9.2°C in these field-collected samples. These findings fit an increased *MdFLC-like* expression after cold treatment in chambers (Porto et al., 2015). The third highest correlation coefficient in correlations with mean temperature was revealed with the expression levels *MdBRR9* (*R* = −0.634, *p* < 0.05). The strongest correlation with temperature showed the expression of *MdPIF4*, however, only after separating the two cultivars (*R* = −0.845 for ‘Gala’, *R* = −0.943 for ‘Pinova’, and *R* = −0.525 all data). This correlation confirms the temperature-sensitive expression variation observed previously (Takeuchi et al., 2018). Also, *PIF4* is well known for its thermoregulatory role in *A. thaliana*, however, mostly for its activation by heat (Kumar et al., 2012). Further study is required to establish, whether allelic differences between ‘Pinova’ and ‘Gala’ *MdPIF4* can confer variation in temperature sensitivity between the two cultivars and whether this difference underlies phenological variation between cultivars.

## Conclusion

**FIGURE 5:**
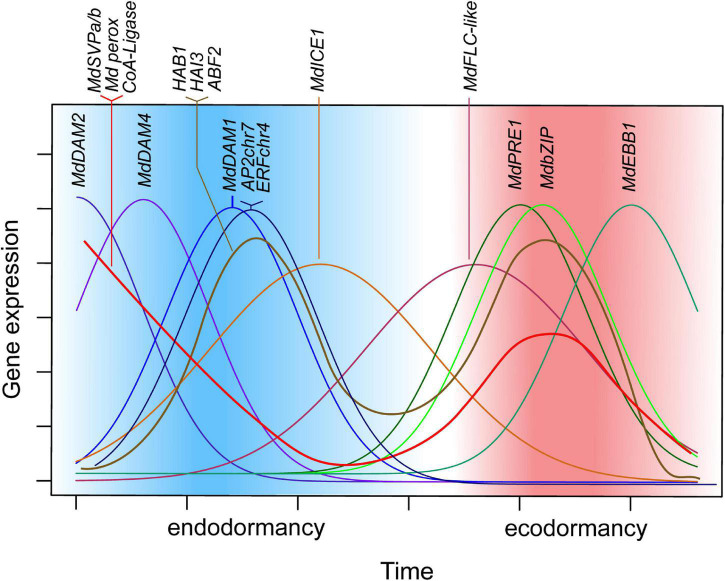
Schematic representation of candidate-gene expression patterns during dormancy progression. Expression of *MdDAM2, MdDAM4*, and *MdDAM1*, as well as *AP2chr7* and *ERFchr4*, occurs in successive order with a single peak during endodormancy. Similarly, during ecodormancy, expression of *MdPRE1, MdbZIP*, and *MdEBB1* show single, consecutive peaks of expression. Expression of *MdSVPa, MdSVPb, MdPeroxidase*, and *CoA-Ligase* is high at the beginning of dormancy and during mid ecodormancy and drops toward phase transitions of endodormancy to ecodormancy and of ecodormancy to bud break. Thus, it appears that multimeric complexes of MdSVPa/b with *MdDAM1*, *MdDAM2*, *MdDAM4*, and *MdFLC-like* can be formed all through dormancy but phase transitions. *MdICE1* and *MdFLC-like* show broader expression curves that also occur in successive order; however, they are both highly expressed during endodormancy to ecodormancy transition.

Gene expression data of dormancy candidate genes were presented, covering the seasons fall, winter, and spring with collected samples every week of two cultivars. In this study, the genes surveyed revealed a set of genes that showed continuous progression of overlapping expression patterns, from paradormancy *via* endodormancy and ecodormancy to bud break (Figure 5). The association of well-established dormancy genes with the distinct phases of dormancy was confirmed; however, evidence was also provided for genes associated with winter dormancy that are less well known for dormancy control. *AP2chr7* and *ERFchr4* expression levels associated with endodormancy, and several genes of the ABA signaling pathway (*HAB1, HAI3*, and *ABF3*) showed a distinct two-peak expression pattern that was revealed through dense sampling. Furthermore, high correlations of expression level with temperature values for *MdICE1, HAB1chr15, MdFLC-like, MdBRR9*, and *MdPIF4* were identified. Interestingly, the slope of the linear correlation of temperature with the expression of *MdPIF4* differed between cultivars. However, whether the two *MdPIF4* alleles differ in their temperature sensitivity needs to be tested further.

## Data Availability Statement

The original contributions presented in the study are included in the article/Supplementary Material, further inquiries can be directed to the corresponding author.

## Author Contributions

JL, AP, and HF designed the research. JL performed the experiments and analyzed the data. JL and HF wrote the manuscript. All authors contributed to the article and approved the submitted version.

## Conflict of Interest

The authors declare that the research was conducted in the absence of any commercial or financial relationships that could be construed as a potential conflict of interest.

## Publisher’s Note

All claims expressed in this article are solely those of the authors and do not necessarily represent those of their affiliated organizations, or those of the publisher, the editors and the reviewers. Any product that may be evaluated in this article, or claim that may be made by its manufacturer, is not guaranteed or endorsed by the publisher.

## References

[B1] Allard A. Bink M. C. Martinez S. Kelner J. J. Legave J. M. di Guardo M. (2016). Detecting QTLs and putative candidate genes involved in budbreak and flowering time in an apple multiparental population. *J. Exp. Bot.* 67 2875–2888. 10.1093/jxb/erw130 27034326 PMC4861029

[B2] Anderson J. L. Richardson E. A. Kesner C. D. (1986). Validation of chill unit and flower bud phenology models for ‘Montmorency’ sour cherry. *Acta Hortic.* 184 71–78.

[B3] Artlip T. McDermaid A. Ma Q. Wisniewski M. (2019). Differential gene expression in nontransgenic and transgenic “M.26” apple overexpressing a peach CBF gene during the transition from eco-dormancy to bud break. *Hortic. Res.* 6:86. 10.1038/s41438-019-0168-9 31666956 PMC6804898

[B4] Bielenberg D. G. Wang Y. Li Z. G. Zhebentyayeva T. Fan S. H. Reighard G. L. (2008). Sequencing and annotation of the evergrowing locus in peach [*Prunus persica* (L.) Batsch] reveals a cluster of six MADS-box transcription factors as candidate genes for regulation of terminal bud formation. *Tree Genet. Genomes* 4 495–507. 10.1007/s11295-007-0126-9

[B5] Bilavcik A. Zamecnik J. Faltus M. (2015). Cryotolerance of apple tree bud is independent of endodormancy. *Front. Plant Sci.* 6:695. 10.3389/fpls.2015.00695 26442012 PMC4561819

[B6] Cattani A. M. da Silveira Falavigna V. Silveira C. P. Buffon V. Dos Santos Maraschin F. Pasquali G. (2020). Type-B cytokinin response regulators link hormonal stimuli and molecular responses during the transition from endo- to ecodormancy in apple buds. *Plant Cell Rep.* 39 1687–1703. 10.1007/s00299-020-02595-z 32959122

[B7] Celton J. M. Martinez S. Jammes M. J. Bechti A. Salvi S. Legave J. M. (2011). Deciphering the genetic determinism of bud phenology in apple progenies: a new insight into chilling and heat requirement effects on flowering dates and positional candidate genes. *New Phytol.* 192 378–392. 10.1111/j.1469-8137.2011.03823.x 21770946

[B8] Chmielewski F.-M. Blümel K. Pálešová I. (2012). Climate change and shifts in dormancy release fordeciduous fruit crops in Germany. *Clim. Res.* 54 209–219.

[B9] Chmielewski F. M. Muller A. Bruns E. (2004). Climate changes and trends in phenology of fruit trees and field crops in Germany, 1961-2000. *Agric. For. Meteorol.* 121 69–78. 10.1016/S0168-1923(03)00161-8

[B10] Cooke J. E. Eriksson M. E. Junttila O. (2012). The dynamic nature of bud dormancy in trees: environmental control and molecular mechanisms. *Plant Cell Environ.* 35 1707–1728. 10.1111/j.1365-3040.2012.02552.x 22670814

[B11] Coville F. V. (1920). The influence of cold in stimulating the growth of plants. *Proc. Natl. Acad. Sci. U.S.A.* 6 434–435. 10.1073/pnas.6.7.434 16576515 PMC1084568

[B12] Daccord N. Celton J. M. Linsmith G. Becker C. Choisne N. Schijlen E. (2017). Highquality de novo assembly of the apple genome and methylome dynamics of early fruit development. *Nat. Genet.* 49 1099–1106. 10.1038/ng.3886 28581499

[B13] Erez A. (2000). “Bud dormancy; phenomenon, problems and solutions in the tropics and subtropics,” in *Temperate Fruit Crops in Warm Climates*, 1 Edn, ed. Erez A. (Dordrecht: Springer Netherlands), 17–48.

[B14] Erez A. Fishman S. Linsley-Noakes G. C. Allan P. (1990). The dynamicmodel for rest completion in peach buds. *Acta Hortic.* 276 165–174.

[B15] Estrella N. Sparks T. H. Menzel A. (2007). Trends and temperature response in the phenology of crops in Germany. *Glob. Change Biol.* 13 1737–1747. 10.1111/j.1365-2486.2007.01374.x

[B16] Fadon E. Fernandez E. Behn H. Luedeling E. (2020). A conceptual framework for winter dormancy in deciduous trees. *Agronomy* 10:241. 10.3390/agronomy10020241

[B17] Falavigna V. D. Porto D. D. Buffon V. Margis-Pinheiro M. Pasquali G. Revers L. F. (2014). Differential transcriptional profiles of dormancy-related genes in apple buds. *Plant Mol. Biol. Report.* 32 796–813. 10.1007/s11105-013-0690-0

[B18] Falavigna V. D. S. Guitton B. Costes E. Andrés F. (2019). I want to (bud) break free: the potential role of DAM and SVP-like genes in regulating dormancy cycle in temperate fruit trees. *Front. Plant Sci.* 9:1990. 10.3389/fpls.2018.01990 30687377 PMC6335348

[B19] Falavigna V. D. S. Severing E. Lai X. Estevan J. Farrera I. Hugouvieux V. (2021). Unraveling the role of MADS transcription factor complexes in apple tree dormancy. *New Phytol.* 232 2071–2088. 10.1111/nph.17710 34480759 PMC9292984

[B20] Faust M. Liu D. H. Millard M. M. Stutte G. W. (1991). Bound versus free-water in dormant apple buds – a theory for endodormancy. *Hortscience* 26 887–890. 10.21273/Hortsci.26.7.887

[B21] Fishman S. Erez A. Couvillon G. A. (1987a). The temperature dependence of dormancy breaking in plants—computer simulation of processes studied under controlled temperatures. *J. Theor. Biol.* 126 309–321.

[B22] Fishman S. Erez A. Couvillon G. A. (1987b). The temperature dependence of dormancy breaking in plants—mathematical analysis of a two-step model involving a cooperative transition. *J. Theor. Biol.* 124 473–483.

[B23] Gilmour S. J. Zarka D. G. Stockinger E. J. Salazar M. P. Houghton J. M. Thomashow M. F. (1998). Low temperature regulation of the *Arabidopsis* CBF family of AP2 transcriptional activators as an early step in cold-induced COR gene expression. *Plant J.* 16 433–442. 10.1046/j.1365-313x.1998.00310.x 9881163

[B24] Heide O. M. (2008). Interaction of photoperiod and temperature in the control of growth and dormancy of *Prunus* species. *Sci. Hortic.* 115 309–314. 10.1016/j.scienta.2007.10.005

[B25] Heide O. M. Prestrud A. K. (2005). Low temperature, but not photoperiod, controls growth cessation and dormancy induction and release in apple and pea. *Tree Physiol.* 25 109–114. 15519992 10.1093/treephys/25.1.109

[B26] Kassambara A. (2017). *Practical Guide to Cluster Analysis in R.* Available online at: http://www.sthda.com: STHDA (accessed November 5, 2021).

[B27] Kotoda N. Hayashi H. Suzuki M. Igarashi M. Hatsuyama Y. Kidou S. (2010). Molecular characterization of FLOWERING LOCUS T-like genes of apple (*Malusx domestica* Borkh.). *Plant Cell Physiol.* 51 561–575. 10.1093/pcp/pcq021 20189942

[B28] Kumar G. Arya P. Gupta K. Randhawa V. Acharya V. Singh A. K. (2016). Comparative phylogenetic analysis and transcriptional profiling of MADS-box gene family identified DAM and FLC-like genes in apple (*Malusx domestica*). *Sci. Rep.* 6:20695. 10.1038/srep20695 26856238 PMC4746589

[B29] Kumar S. V. Lucyshyn D. Jaeger K. E. Alós E. Alvey E. Harberd N. P. (2012). Transcription factor PIF4 controls the thermosensory activation of flowering. *Nature* 484 242–245. 10.1038/nature10928 22437497 PMC4972390

[B30] Lang G. A. (1987). Dormancy: a new universal terminology. *Hortic. Sci.* 22 817–820.

[B31] Lee J. H. Jung J. H. Park C. M. (2015). INDUCER OF CBF EXPRESSION 1 integrates cold signals into FLOWERING LOCUS C-mediated flowering pathways in *Arabidopsis*. *Plant J.* 84 29–40. 10.1111/tpj.12956 26248809

[B32] Li J. Z. Yan X. H. Ahmad M. Yu W. J. Song Z. Z. Ni J. B. (2021). Alternative splicing of the dormancy-associated MADS-box transcription factor gene PpDAM1 is associated with flower bud dormancy in ‘Dangshansu’ pear (*Pyrus pyrifolia* white pear group). *Plant Physiol. Biochem.* 166 1096–1108. 10.1016/j.plaphy.2021.07.017 34304127

[B33] Luedeling E. Zhang M. H. Girvetz E. H. (2009). Climatic changes lead to declining winter chill for fruit and nut trees in California during 1950-2099. *PLoS One* 4:e6166. 10.1371/journal.pone.0006166 19606220 PMC2707005

[B34] Maechler M. Rousseeuw P. Struyf A. Hubert M. Hornik K. (2021). *cluster: Cluster Analysis Basics and Extensions [Online].* Available online at: https://CRAN.R-project.org/package=cluster (accessed November 5, 2021).

[B35] Malagi G. Sachet M. R. Citadin I. Herter F. G. Bonhomme M. Regnard J. L. (2015). The comparison of dormancy dynamics in apple trees grown under temperate and mild winter climates imposes a renewal of classical approaches. *Trees Struct. Funct.* 29 1365–1380. 10.1007/s00468-015-1214-3

[B36] Maurya J. P. Bhalerao R. P. (2017). Photoperiod- and temperature-mediated control of growth cessation and dormancy in trees: a molecular perspective. *Ann. Bot.* 120 351–360. 10.1093/aob/mcx061 28605491 PMC5591416

[B37] Milyaev A. Kofler J. Klaiber I. Czemmel S. Pfannstiel J. Flachowsky H. (2021). Toward systematic understanding of flower bud induction in apple: a multi-omics approach. *Front. Plant Sci.* 12:604810. 10.3389/fpls.2021.604810 33841452 PMC8030266

[B38] Mimida N. Saito T. Moriguchi T. Suzuki A. Komori S. Wada M. (2015). Expression of DORMANCY-ASSOCIATED MADS-BOX (DAM)-like genes in apple. *Biol. Plant.* 59 237–244. 10.1007/s10535-015-0503-4

[B39] Miotto Y. E. Tessele C. Czermainski A. B. C. Porto D. D. Falavigna V. D. S. Sartor T. (2019). Spring is coming: genetic analyses of the bud break date locus reveal candidate genes from the cold perception pathway to dormancy release in apple (*Malusx domestica* Borkh.). *Front. Plant Sci.* 10:33. 10.3389/fpls.2019.00033 30930909 PMC6423911

[B40] Moser M. Asquini E. Miolli G. V. Weigl K. Hanke M. V. Flachowsky H. (2020). The MADS-box gene MdDAM1 controls growth cessation and bud dormancy in apple. *Front. Plant Sci.* 11:1003. 10.3389/fpls.2020.01003 32733512 PMC7358357

[B41] Nishiyama S. Matsushita M. C. Yamane H. Honda C. Okada K. Tamada Y. (2021). Functional and expressional analyses of apple FLC-like in relation to dormancy progress and flower bud development. *Tree Physiol.* 41 562–570. 10.1093/treephys/tpz111 31728534

[B42] Pfaffl M. W. (2001). A new mathematical model for relative quantification in real-time RT-PCR. *Nucleic Acids Res.* 29:e45. 10.1093/nar/29.9.e45 11328886 PMC55695

[B43] Porto D. D. Bruneau M. Perini P. Anzanello R. Renou J. P. dos Santos H. P. (2015). Transcription profiling of the chilling requirement for bud break in apples: a putative role for FLC-like genes. *J. Exp. Bot.* 66 2659–2672. 10.1093/jxb/erv061 25750421

[B44] Quesada-Traver C. Guerrero B. I. Badenes M. L. Rodrigo J. Rios G. Lloret A. (2020). Structure and expression of bud dormancy-associated MADS-box genes (DAM) in European *Plum*. *Front. Plant Sci.* 11:1288. 10.3389/fpls.2020.01288 32973847 PMC7466548

[B45] Reim S. Rohr A. D. Winkelmann T. Weiss S. Liu B. Y. Beerhues L. (2020). Genes involved in stress response and especially in phytoalexin biosynthesis are upregulated in four malus genotypes in response to apple replant disease. *Front. Plant Sci.* 10:1724. 10.3389/fpls.2019.01724 32180775 PMC7059805

[B68] Richardson E. A. Seeley S. D. Walker D. R. (1974). A model for estimating the completion of rest for “Redhaven” and “Elberta” peach trees. *Hortic Sci.* 9, 331–332.

[B46] Rothkegel K. Sanchez E. Montes C. Greve M. Tapia S. Bravo S. (2017). DNA methylation and small interference RNAs participate in the regulation of MADS-box genes involved in dormancy in sweet cherry (*Prunus avium* L.). *Tree Physiol.* 37 1739–1751. 10.1093/treephys/tpx055 28541567

[B47] Saure M. C. (1985). “Dormancy release in desciduous fruit trees,” in *Horticultural Reviews*, ed. Janick J. (Westport, CT: Wiley).

[B48] Singh R. K. Svystun T. AlDahmash B. Jönsson A. M. Bhalerao R. P. (2017). Photoperiod- and temperature-mediated control of phenology in trees - a molecular perspective. *New Phytol.* 213 511–524. 10.1111/nph.14346 27901272

[B49] Takemura Y. Kuroki K. Shida Y. Araki S. Takeuchi Y. Tanaka K. (2015). Comparative transcriptome analysis of the less-dormant Taiwanese pear and the dormant Japanese pear during winter season. *PLoS One* 10:e0139595. 10.1371/journal.pone.0139595 26451604 PMC4599857

[B50] Takeuchi T. Matsushita M. C. Nishiyama S. Yamane H. Banno K. Tao R. (2018). RNA-sequencing analysis identifies genes associated with chilling-mediated endodormancy release in apple. *J. Am. Soc. Hort. Sci.* 143:194. 10.21273/jashs04345-18

[B51] Tanino K. K. Kalcsits L. Silim S. Kendall E. Gray G. R. (2010). Temperature-driven plasticity in growth cessation and dormancy development in deciduous woody plants: a working hypothesis suggesting how molecular and cellular function is affected by temperature during dormancy induction. *Plant Mol. Biol.* 73 49–65. 10.1007/s11103-010-9610-y 20191309

[B52] Tuan P. A. Bai S. L. Saito T. Imai T. Ito A. Moriguchi T. (2016). Involvement of EARLY BUD-BREAK, an AP2/ERF transcription factor gene, in bud break in Japanese pear (*Pyrus pyrifolia* Nakai) lateral flower buds: expression, histone modifications and possible target genes. *Plant Cell Physiol.* 57 1038–1047. 10.1093/pcp/pcw041 26940832

[B53] Urrestarazu J. Muranty H. Denancé C. Leforestier D. Ravon E. Guyader A. (2017). Genome-wide association mapping of flowering and ripening periods in apple. *Front. Plant Sci.* 8:1923. 10.3389/fpls.2017.01923 29176988 PMC5686452

[B54] van Dyk M. M. Soeker M. K. Labuschagne I. F. Rees D. J. G. (2010). Identification of a major QTL for time of initial vegetative budbreak in apple (*Malus x domestica* Borkh.). *Tree Genet. Genomes* 6 489–502.

[B55] Vimont N. Fouché M. Campoy J. A. Tong M. Arkoun M. Yvin J. C. (2019). From bud formation to flowering: transcriptomic state defines the cherry developmental phases of sweet cherry bud dormancy. *BMC Genomics* 20:974. 10.1186/s12864-019-6348-z 31830909 PMC6909552

[B56] Wei T. Simko V. (2021). *R Package ‘Corrplot’: Visualization of a Correlation Matrix [Online].* Available online at: https://github.com/taiyun/corrplot (accessed April 10, 2021).

[B57] Weinberger J. H. (1950). Chilling requirements of peach varieties. *J. Am. Soc. Hort. Sci.* 56 11–16.

[B58] Wickham H. (2016). *ggplot2: Elegant Graphics for Data Analysis.* New York, NY: Springer-Verlag.

[B59] Willige B. C. Zander M. Yoo C. Y. Phan A. Garza R. M. Trigg S. A. (2021). PHYTOCHROME-INTERACTING FACTORs trigger environmentally responsive chromatin dynamics in plants. *Nat. Genet.* 53 955–961. 10.1038/s41588-021-00882-3 34140685 PMC9169284

[B60] Wisniewski M. Norelli J. Artlip T. (2015). Overexpression of a peach CBF gene in apple: a model for understanding the integration of growth, dormancy, and cold hardiness in woody plants. *Front. Plant Sci.* 6:85. 10.3389/fpls.2015.00085 25774159 PMC4343015

[B61] Wu R. Cooney J. Tomes S. Rebstock R. Karunairetnam S. Allan A. C. (2021). RNAimediated repression of dormancy-related genes results in evergrowing apple trees. *Tree Physiol.* 41 1510–1523. 10.1093/treephys/tpab007 33564851

[B62] Wu R. Tomes S. Karunairetnam S. Tustin S. D. Hellens R. P. Allan A. C. (2017). SVP-like MADS box genes control dormancy and budbreak in apple. *Front. Plant Sci.* 8:477. 10.3389/fpls.2017.00477 28421103 PMC5378812

[B63] Xie F. Xiao P. Chen D. Xu L. Zhang B. (2012). miRDeepFinder: a miRNA analysis tool for deep sequencing of plant small RNAs. *Plant Mol. Biol.* 10.1007/s11103-012-9885-2 22290409

[B64] Yordanov Y. S. Ma C. Strauss S. H. Busov V. B. (2014). EARLY BUD-BREAK 1 (EBB1) is a regulator of release from seasonal dormancy in poplar trees. *Proc. Natl. Acad. Sci. U.S.A.* 111 10001–10006. 10.1073/pnas.1405621111 24951507 PMC4103365

[B65] Yu J. Conrad A. O. Decroocq V. Zhebentyayeva T. Williams D. E. Bennett D. (2020). Distinctive gene expression patterns define endodormancy to ecodormancy transition in apricot and peach. *Front. Plant Sci.* 11:180. 10.3389/fpls.2020.00180 32180783 PMC7059448

[B66] Zhao K. Zhou Y. Z. Ahmad S. Xu Z. D. Li Y. S. Yang W. R. (2018). Comprehensive cloning of *Prunus mume* dormancy associated MADS-box genes and their response in flower bud development and dormancy. *Front. Plant Sci.* 9:17. 10.3389/fpls.2018.00017 29449849 PMC5800298

[B67] Zhu H. Chen P. Y. Zhong S. L. Dardick C. Callahan A. An Y. Q. (2020). Thermalresponsive genetic and epigenetic regulation of DAM cluster controlling dormancy and chilling requirement in peach floral buds. *Hortic. Res.* 7:114. 10.1038/s41438-020-0336-y 32821397 PMC7395172

