# Erratum: Time-Resolved Analysis of Candidate Gene Expression and Ambient Temperature During Bud Dormancy in Apple

**DOI:** 10.3389/fpls.2022.930169

**Published:** 2022-05-24

**Authors:** Frontiers Production Office

**Affiliations:** Frontiers Media SA, Lausanne, Switzerland

**Keywords:** apple, dormancy, temperature, DAM genes, endodormancy, ecodormancy

Due to a production error, the reference for “(Falavigna et al., 2021)” was incorrectly written as “(Chmielewski et al., 2012).” A correction has been made to the **Introduction**, paragraph 5:

“*Short Vegetative Phase* (*SVP*) genes are structurally similar to *DAM* genes and are also important for dormancy control. Lines with transgenically downregulated levels of *MdDAM* and *MdSVP* genes are not able to get into the dormant state (Wu et al., 2017, 2021; Moser et al., 2020). Interestingly, *MdDAM, MdSVP*, and *Md Flowering Locus C-like* (*MdFLC*-like) genes form multimeric complexes that bind to specific DNA regions (Falavigna et al., 2021)”.

The publisher apologizes for this mistake. The original article has been updated.
