# Supplementary figures and images for: Time-Resolved Analysis of Candidate Gene Expression and Ambient Temperature During Bud Dormancy in Apple
Source: Front Plant Sci. 2022 Jan 17;12:803341. doi: 10.3389/fpls.2021.803341 (PMC8802299; doi:10.3389/fpls.2021.803341)

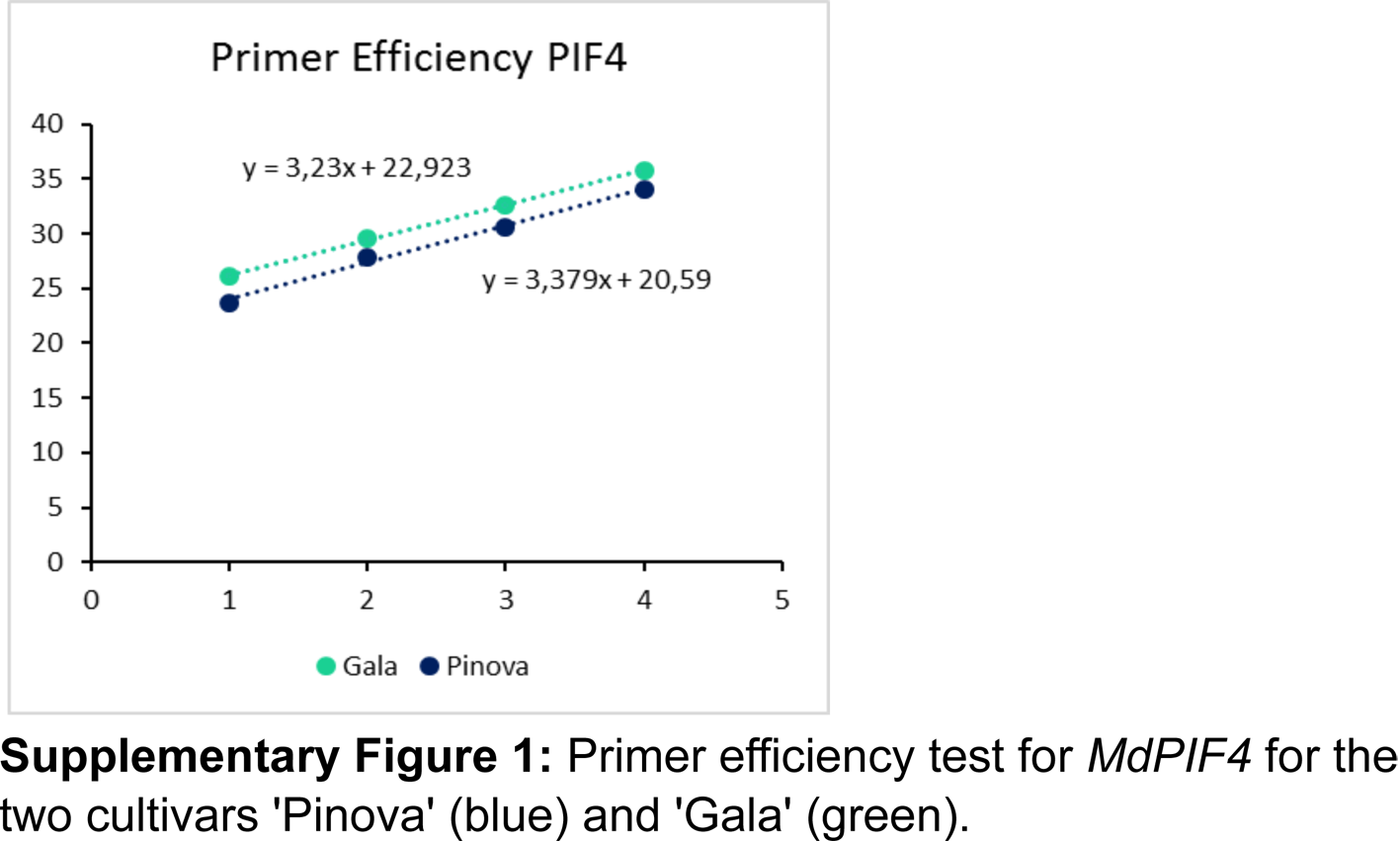

Supplement: Supplementary file 4 [file Image_1.TIF]

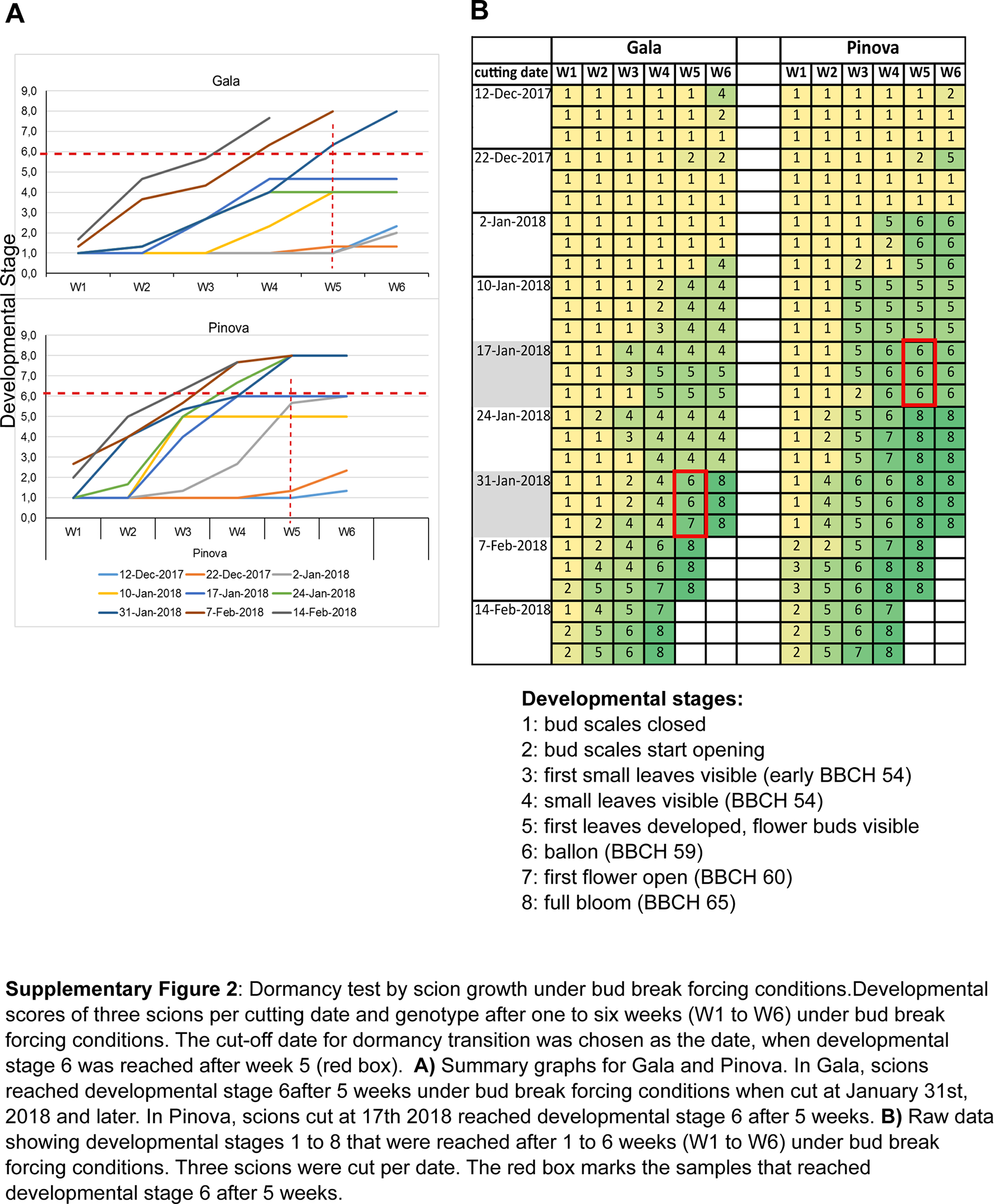

Supplement: Supplementary file 5 [file Image_2.TIF]

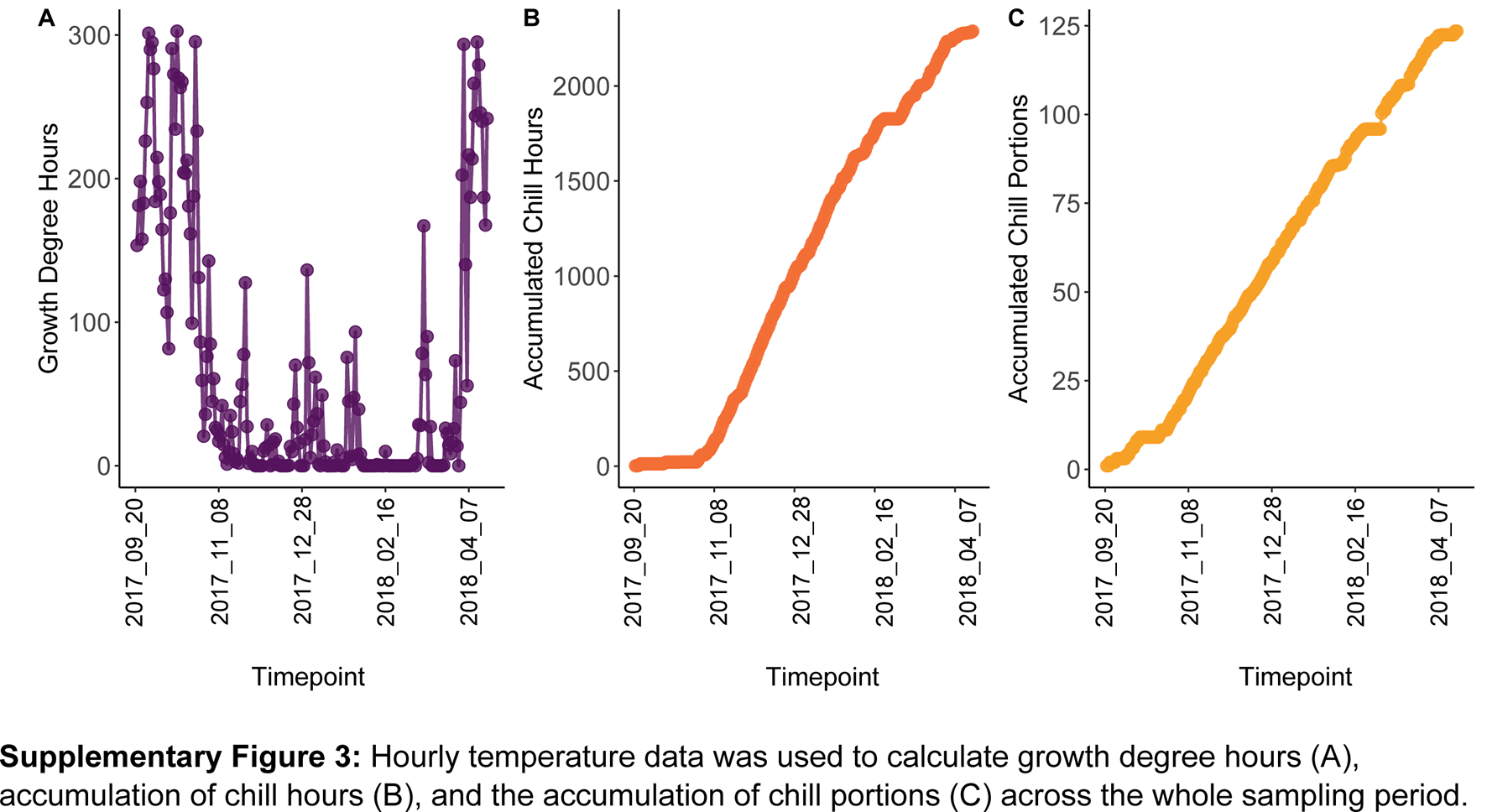

Supplement: Supplementary file 6 [file Image_3.TIF]

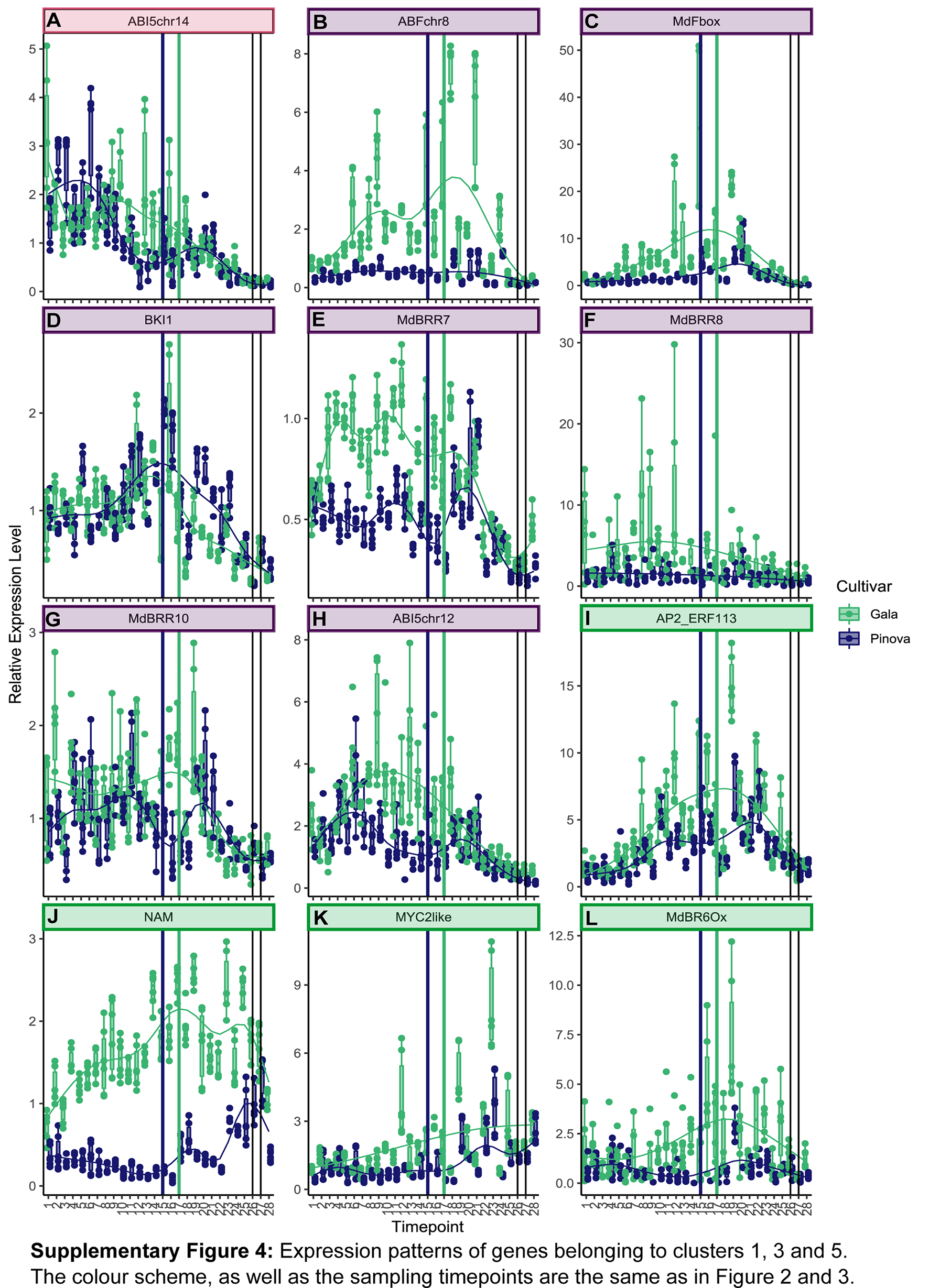

Supplement: Supplementary file 7 [file Image_4.TIF]

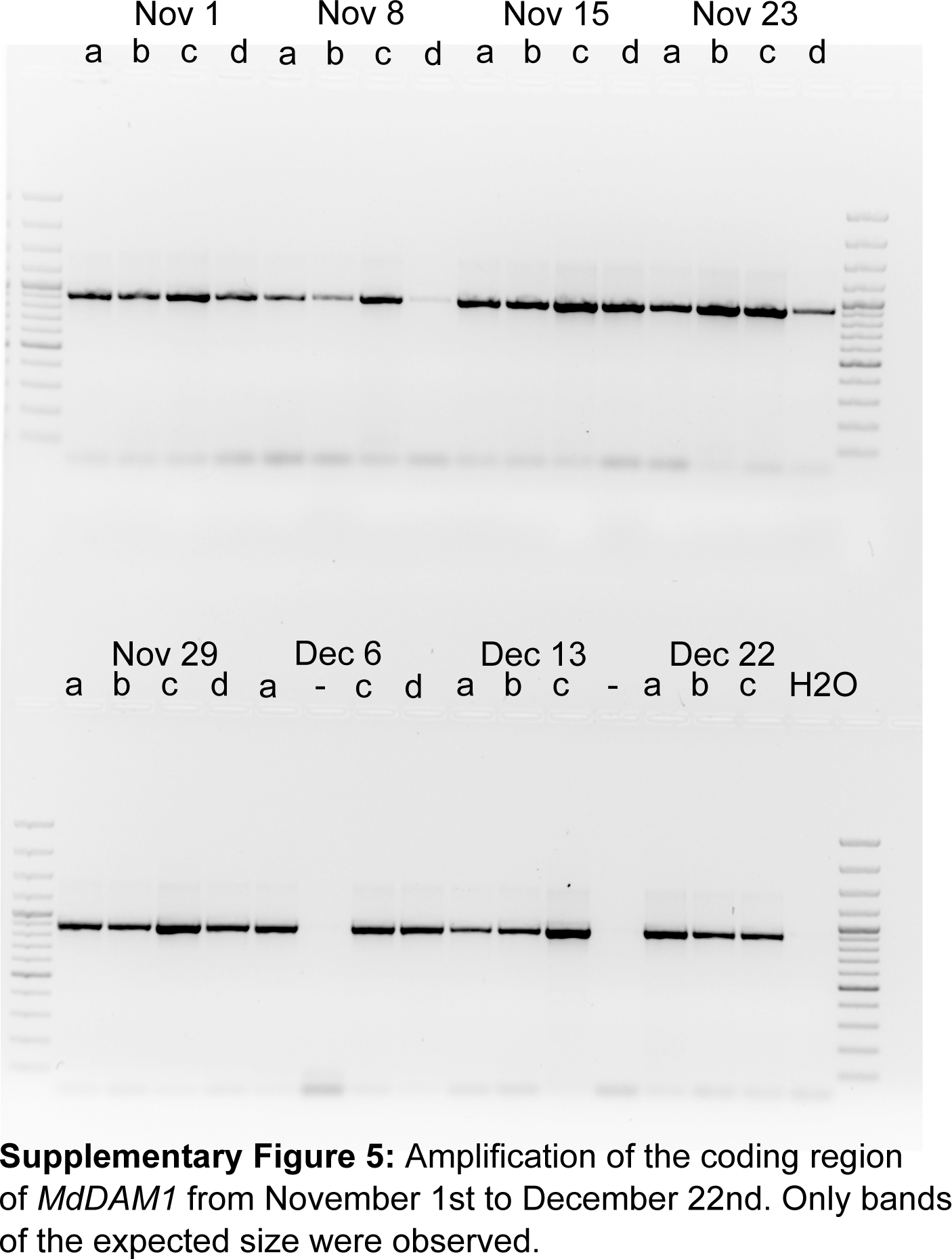

Supplement: Supplementary file 8 [file Image_5.TIF]

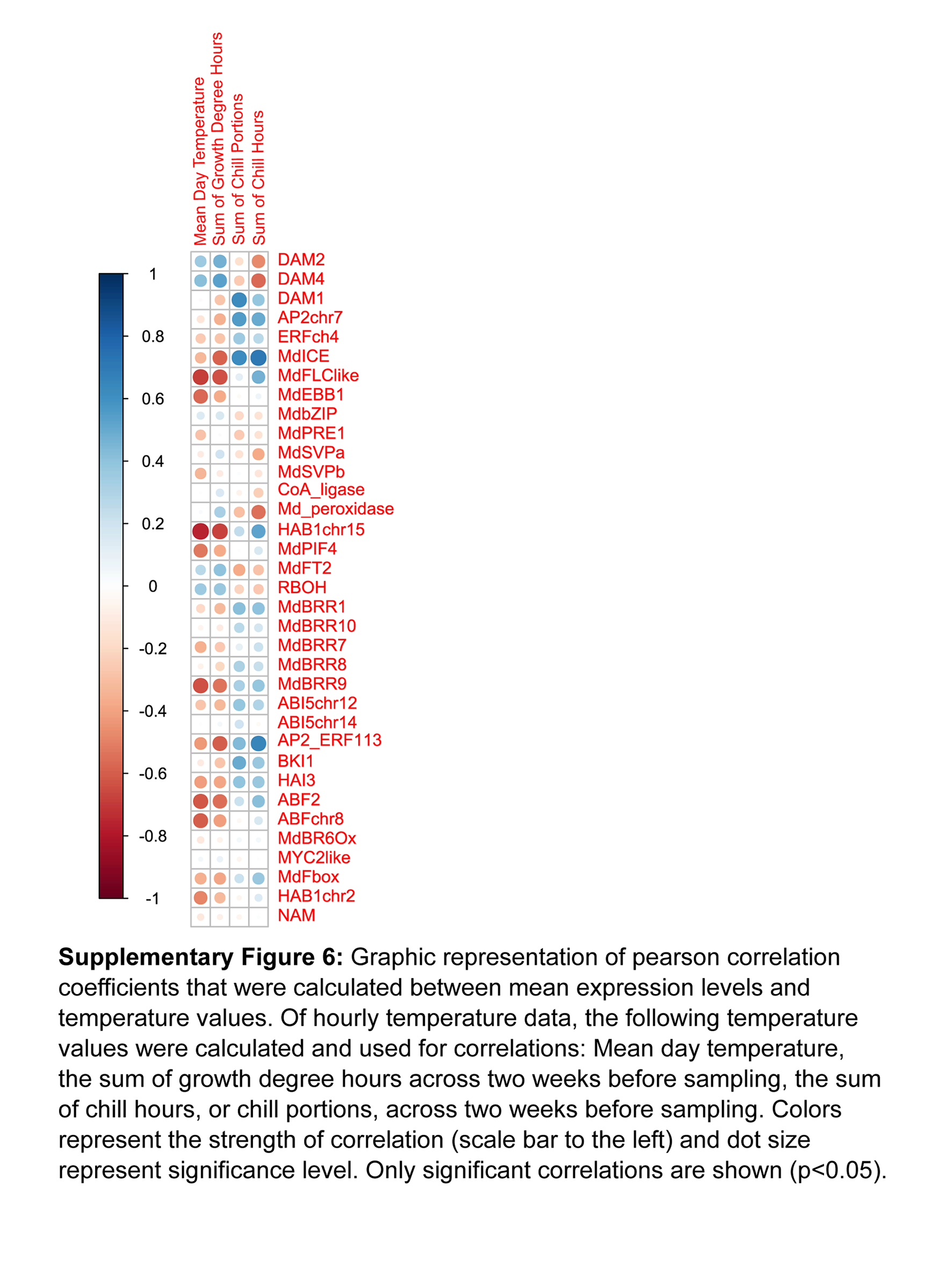

Supplement: Supplementary file 9 [file Image_6.TIF]
